# Longitudinal Effects of Adolescent Digital Media Use on Mental Health in Young Adulthood †

**DOI:** 10.3390/children13020215

**Published:** 2026-02-01

**Authors:** Caroline S. Watson, Christopher C. Henrich, Dustin M. Long, Aaron D. Fobian

**Affiliations:** 1 Department of Psychology, University of Alabama at Birmingham, Birmingham, AL 35233, USA; 2 Department of Biostatistics and Data Science, Wake Forest University, Winston-Salem, NC 27109, USAafobian@uabmc.edu (A.D.F.)

**Keywords:** adolescent, mental health, media exposure, young adulthood

## Abstract

Background/Objectives: Research on the relationship between digital media use in adolescence and mental health outcomes in young adulthood remains unclear. This study aims to (1) assess how trajectories of digital media use from adolescence to young adulthood predict mental health outcomes and (2) identify factors in adolescence that contribute to digital media use trajectories. Methods: Participants (M_age_ = 15.53 years; 56.86% female; 66.89% White) from the National Longitudinal Study of Adolescent and Adult Health database provided digital media use data across Waves I–IV. At Wave I, participants self-reported parental support, family connectedness, face-to-face interactions with peers, and self-esteem. At Wave IV, participants self-reported anxiety and depression diagnoses, depressive symptomology, suicidal ideation and attempts, and short-term and working memory. General linear and logistic regression models assessed the relationships. Results: Four trajectory groups emerged: Group 1 “increase” (9.97%), Group 2 “low” (73.36%), Group 3 “decrease” (13.94%), and Group 4 “high” (2.73%). Individuals in Group 4 experienced decreased short-term memory compared to individuals in Group 2. The odds of a suicide attempt in the past 12 months were significantly higher for individuals in Groups 3 and 4 compared to Group 2. Conclusions: Patterns of digital media use from adolescence to young adulthood may contribute to suicide attempts and short-term memory in young adulthood, highlighting the need for interventions to reduce screen time. Non-significant findings highlight the need for additional research aimed at clarifying these relationships and identifying factors in early adolescence that may contribute to digital media use trajectories.

## 1. Introduction

Adolescence is a critical period for developing healthy habits, including those related to digital media use [1,2]. Accordingly, the American Academy of Pediatrics recommends adolescents spend no more than 2 h per day on digital media [1]. However, in 2021, American adolescents spent 8 h and 39 min per day on digital media, which is a 77-min increase since 2019 and 119-min increase since 2015 [3,4]. As digital media use among adolescents has increased, there has been a sharp decline in adolescent mental health. Large scale screening studies on the mental health of adolescents show drastic increases in anxiety and depressive symptoms, major depressive episodes, suicidal ideation, and suicide attempts since 2010 [5]. There is concern that such decline in mental health may be associated with increased digital media use [5,6]. However, research on the associations between digital media use and mental health outcomes among adolescents, including depression and anxiety disorders, depressive symptomology, suicidality, and cognitive health, has found conflicting results [7].

Anxiety disorders are the most prevalent mental health disorders among adolescents [8]. Adolescent anxiety poses several long-term health risks, including poor mental health and adverse physical health conditions in adulthood [9,10,11]. Increasingly, evidence suggests that digital media use may contribute to adolescent anxiety. For example, digital media use may reduce the frequency of adolescents’ face-to-face interactions with peers, which may result in decreased feelings of closeness. Considering the importance of meaningful peer relationships in adolescence, decreased feelings of closeness may exacerbate anxiety [7]. However, cross-sectional and longitudinal research on the relationship between digital media use and anxiety has found conflicting results [11,12]. One study of 2525 adolescents found that adolescents who spent less than four hours per day on digital media were less likely to experience symptoms of anxiety disorders (i.e., generalized anxiety disorder, social phobia, and panic disorder) 12 months later than adolescents who spent more than four hours per day on digital media [13]. Conversely, a 2016 study that followed adolescents for 11 years found no relationship between digital media use and anxiety. However, this study only examined television and computer games in their measure of digital media use [14].

The relationship between digital media use and depression is also unclear. Several studies have demonstrated positive correlations between digital media use and depression and depressive symptoms [15]. One longitudinal study found that each additional hour spent viewing a screen during adolescence was associated with a 1.58 times greater risk for depression in young adulthood [16]. In a study of 14–17-year-olds, adolescents who used digital media more than 7 h a day were more than two times more likely to be diagnosed with depression [7]. The sedentary nature of digital media use may explain this relationship, as sedentary behaviors are consistently associated with increased risk for depression and depressive symptomology [17]. Conversely, a substantial amount of cross-sectional research suggests little or no relationship between digital media use and depression [18,19]. In a recent literature review, only 45% of studies included found associations between digital media use and depressive symptoms [14].

The relationship between digital media use and suicide attempts and suicidal ideation (i.e., thoughts of or plans to commit suicide) among adolescents is especially concerning [5,6]. Since 2010, emergency hospital visits for suicide attempts and suicidal ideation among adolescents have nearly doubled. Among 10–12-year-old girls, self-poisoning rates have quadrupled, hospital admissions for self-harm have tripled, and suicide rates have doubled [5]. Increased digital media use is often blamed for this marked increase [6]. A recent literature review found consistent evidence linking time spent using digital media to suicidal ideation among adolescents in Western countries [20]. This relationship may be due to depression, as adolescents who are more depressed are at increased risk for suicidal ideation and suicide attempts and often spend more time on digital media [6]. Cyberbullying via digital media may also play a role in this relationship, as victims of cyberbullying are two times more likely to attempt suicide [21]. The media contagion effect—the idea that exposure to suicide through media can increase individual risk for suicidal ideation and suicide attempts—may also contribute to the relationship between digital media use and suicide [21,22,23]. Research consistently reports associations between media reports of suicide and increased suicide rates [23]. One study found that 15–19-year-olds exposed to a death by suicide in their county were two to four times more likely to commit suicide [22].

Recent research has also considered the effects of digital media use on cognitive ability. In a cross-sectional study of 11,875 adolescents, adolescents who exceeded 7 h of digital media use per day were 40% less likely to achieve higher grades in school [12]. Similarly, a recent systematic review found consistent evidence relating increased digital media use to slowed learning and acquisition [24]. Conversely, a systematic review and meta-analysis of 58 cross-sectional studies found no association between digital media use and academic performance across studies [25]. However, most studies have focused on academic performance and learning ability as measures of cognitive ability, rather than assessing specific aspects of cognitive functioning such as working memory and short-term memory.

Overall, the relationship between digital media use and mental health remain unclear. Existing research has largely relied on cross-sectional designs and single time-point assessments of digital media use, which limit conclusions about temporal ordering, whereas cumulative patterns of use across adolescence may better reflect the relationships between digital media use and mental health outcomes. Longitudinal studies that will allow deeper insight into these relationships are needed. Furthermore, it is important to identify factors in adolescence that may contribute to digital media use, including parental support, face-to-face interactions with peers, family connectedness, and self-esteem.

Digital media use may be influenced by parenting behaviors [26]. The previous literature reports that children of more involved parents spend less time on digital media, and adolescents who spend more time on digital media report poorer parental attachment [27,28]. Within the family system, digital media use has been associated with poorer family relationships and less time spent together as a family [28]. Similarly, digital media use has been associated with poor attachment to peers, possibly due to increased time spent on digital media and decreased time spent face-to-face with peers [7,28]. Research has yet to identify the direction of these relationships and how parental support, face-to-face interactions with peers, and family connectedness may contribute to digital media use.

To address these gaps in the literature, we sought to assess how patterns of digital media use over time (i.e., trajectories) across the adolescent transition to adulthood are associated with mental health outcomes in young adulthood, including short-term memory, working memory, diagnoses of depression and anxiety disorders, depressive symptomology, and suicidality. We further sought to assess how parental support, face-to-face interactions with peers, family connectedness, and self-esteem in adolescence are associated with digital media use across the transition from adolescence into young adulthood. Overall, we aimed to provide a clearer understanding of the long-term effects of digital media use and identify factors in adolescence that predict digital media use. We hypothesized that adolescents with increasing or consistently high amounts of digital media use would experience poorer mental health outcomes in young adulthood, and that adolescents with lower parental support, family connectedness, and self-esteem and fewer face-to-face interactions with peers would exhibit increasing or consistently high digital media use over time.

## 2. Materials and Methods

A secondary analysis of data from the National Longitudinal Study of Adolescent Health (Add Health) database was conducted and approved by the University of Alabama at Birmingham. Add Health is a nationally representative, longitudinal study designed to examine the social, behavioral, and biological determinants of health across adolescence to young adulthood. Participants were recruited using a multistage, school-based sampling design that included students from 80 high schools across the United States. Accordingly, the Add Health database provides a racially and ethnically diverse sample, including White (52.00%), Black, (22.20%), Mexico (8.50%), Central South America (3.10%), Philippines (3.10%), Other Asia (2.90%), Puerto Rico (2.80%), Cuba (2.50%), China (1.70%), Native American (1.20%). Data collection involved standardized, structured questionnaires administered by trained interventions through a combination of in-home and in-school interviews and questionnaires administered to participants as they transitioned from adolescence into young adulthood. In-home interviews lasted approximately 90 min and included both interviewer-administered and self-administered components. We focused on digital media use questions asked in Waves I–IV as predictors of mental health outcomes in Wave IV. Study methods and results are reported following the Strengthening the Reporting of Observational Studies in Epidemiology (STROBE) statement for cross-sectional studies.

### 2.1. Participants

Participants who responded to digital media use questions asked in Waves I–IV were included. To maintain the goal of following participants across the transition from adolescence into young adulthood, participants older than 17 years of age at Wave I were excluded from analyses. Participants were aged 11–17 at Wave I (1994) and 24–30 at Wave IV (2008–2009). Participants who were pregnant at Wave IV were also excluded from analyses. The final sample included 6767 participants (M_age_ = 15.53, SD = 1.42; 56.86% female, 66.89% White). 

### 2.2. Measures

#### 2.2.1. Demographics

At Wave I, adolescents provided self-reports of sex, date of birth, and race, and parents provided self-reports of total household income before taxes.

#### 2.2.2. Digital Media Use

In Waves I–III, participants were asked, “How many hours a week do you play video or computer games?”, “How many hours a week do you watch television?”, and “How many hours a week do you watch videos?”. At Wave IV, participants were asked “How many hours a week do you play video or computer games?”, “In the past seven days, how many hours did you spend using the internet, for example, accessing your email or using the web?”, and “In the past 7 days, how many hours did you watch television or videos, including VHS, DVDs, or music videos?”. Responses were recorded in total hours and summed to construct a single measure of digital media use per week at each wave.

#### 2.2.3. Pregnancy Status

At Wave IV, participants provided self-reports of pregnancy status by responding “yes/no” to the question, “Are you pregnant now?” Participants who reported current pregnancy status at Wave IV were excluded from the study.

#### 2.2.4. Rurality

At Wave I, interviewers were asked to describe the immediate area or street where the respondent lived, including “rural”, “suburban”, “urban (residential only)”, “3 or more commercial properties (mostly retail)”, “3 or more commercial properties (mostly wholesale or industrial)”, and “other”. At Wave IV, interviewers were again asked to describe the immediate area or street where the respondent lived, including “rural farm”, “rural town”, “suburban”, “urban (residential only)”, “3 or more commercial properties (mostly retail)”, “3 or more commercial properties (mostly wholesale or industrial)”, and “other”.

#### 2.2.5. Depression

Depression diagnosis at Wave IV was determined using the question, “Have you ever been diagnosed with depression?” with a “yes/no” response.

#### 2.2.6. Depressive Symptomology

At Wave IV, depressive symptomology was determined using a modified scale from the Center for Epidemiological Studies—Depression scale. The prior literature demonstrates high internal consistency among males (α = 0.84) and females (α = 0.89) in the Add Health cohort [29] and across racial and ethnic groups [30]. Participants were asked how often the following statements were true in the past week: “You felt depressed”, “You enjoyed life”, “You felt sad”, “You felt that people disliked you”, “You felt that you could not shake off the blues, even with help from your family and your friends”, “You felt that you were too tired to do things”, “You felt you were just as good as other people”, “You had trouble keeping your mind on what you were doing”, and “You were bothered by things that don’t usually bother you”. Responses were recorded using a 4-point Likert scale with response labels “never to rarely”, “sometimes”, “a lot of the time”, and “most of the time or all of the time”. Responses marked as “sometimes”, “a lot of the time”, and “most of the time or all of the time” were classified as depressive symptomology, and responses marked as “never to rarely” were classified as no depressive symptomology. From this information, a total depressive symptomology score was calculated by summing the total number of items classified as depressive symptomology for a final score ranging from 0 to 9.

#### 2.2.7. Anxiety

At Wave IV, anxiety diagnosis was determined using the question, “Has a doctor, nurse, or other health care provider ever told you that you have or had anxiety or panic disorder?” Participants provided a “yes/no” response.

#### 2.2.8. Suicidal Ideation and Suicide Attempts

At Wave IV, participants were asked, “During the past 12 months, did you ever seriously think about committing suicide?” and “During the past 12 months, how many times did you attempt suicide?” Response values were recorded individually as suicidal ideation and suicide attempts, respectively.

#### 2.2.9. Cognitive Health

At Wave IV, participants completed two cognitive tasks assessing working memory and short-term memory. In the working memory task, participants were read a set of numbers and asked to repeat the numbers in reverse order. Each number set included two number sequences of the same length, and the length of numbers in the sequence increased by one number with each set. The task ended when the participant failed to accurately repeat both trials of the same length or accurately completed all seven sets. One point was awarded for each number set accurately completed and summed to construct a single measure of working memory at Wave IV. In the short-term memory task, participants were read 15 words and asked to recall as many words as they could remember. The task ended when the participant failed to recall any more words or after 90 s. One point was awarded for each word accurately recalled and summed to construct a single measure of short-term memory at Wave IV. Both measures of cognitive health have been validated against tests of cognition performance and measures of brain health [31].

#### 2.2.10. Self-Esteem

At Wave I, self-esteem was determined using a modified version of the Rosenberg Self-Esteem Inventory. The prior literature demonstrates strong validity among males (α = 0.83) and females (α = 0.84) in the Add Health cohort [29]. Participants were asked how much they agreed or disagreed with the following statements: “You feel like you are doing just about everything right”, “You feel loved and wanted”, “You feel socially accepted”, “You have lots of good qualities”, “You have lots to be proud of”, and “You like yourself just the way you are”. Responses were recorded using a 5-point Likert scale with response labels “strongly agree”, “agree”, “neither agree nor disagree”, “disagree”, and “strongly disagree”. Responses marked as “strongly agree” and “agree” were classified as presence of self-esteem, and responses marked as “neither agree nor disagree”, “disagree”, and “strongly disagree” were not classified as presence of self-esteem. A total self-esteem score was calculated by summing the total number of items classified as presence of self-esteem for a final score ranging from 0 to 6.

#### 2.2.11. Parental Support

At Wave I, participants responded to a series of questions regarding their in-house (biological, step, adoptive, or foster) mother and/or father. Participants with both an in-house mother and father provided responses on their mother and father, and participants with one in-house mother or father provided responses only on their in-house mother or father. Participants were asked, “How close do you feel to your (biological, step, adoptive, or foster) mother/father?” and “How much do you think your (biological, step, adoptive, or foster) mother/father cares about you?”. Responses were recorded on a 5-point Likert scale with response labels “not at all”, “a little”, “some”, “quite a bit”, and “very much”. Participants were also asked how much they agreed or disagreed with the following statements: “Most of the time your mother/father is warm and loving toward you”, “You are satisfied with the way your mother/father and you communicate”, and “Overall, you are satisfied with your relationship with your mother/father”. Responses were recorded using a 5-point Likert scale with response labels “strongly agree”, “agree”, “neither agree nor disagree”, “disagree”, and “strongly disagree”. Responses marked as “a little”, “some”, “quite a bit”, “very much”, “strongly agree”, or “agree” were classified as parental support, and responses marked as “not at all”, “neither agree nor disagree”, “disagree,” and “strongly disagree” were not classified as parental support. In cases where both mother and father data were available, responses classified as parental support were summed for the mother and father individually, then averaged to construct a single measure of parental support at Wave I for a final score ranging from 0 to 5. In cases where only mother or father data were available, responses classified as parental support for the mother or father were summed to construct a single measure of parental support at Wave I for a final score ranging from 0 to 5. Thus, data that were missing for legitimate reasons (e.g., single parent) were not excluded to retain maximum sample size.

#### 2.2.12. Family Connectedness

At Wave I, participants were asked, “How much do you feel that people in your family understand you?”, “How much do you feel that you and your family have fun together?”, and “How much do you feel that your family pays attention to you?”. Responses were recorded on a 5-point Likert scale with response labels “not at all”, “very little”, “somewhat”, “quite a bit”, and “very much”. Responses marked as “very little”, “somewhat”, “quite a bit”, and “very much” were classified as family closeness, and responses marked as “not at all” were not classified as family closeness. Responses classified as family closeness were summed to construct a single measure of family closeness for participants at Wave I for a final score ranging from 0 to 3. The prior literature suggests strong psychometric properties among measures of family context in the Add Health database [32].

### 2.3. Data Analyses

A group-based modeling technique (PROC TRAJ; assuming a censored normal distribution) was used to identify latent trajectory classes of digital media use across Waves I–IV. This approach was selected to examine whether distinct patterns of digital media use overtime, particularly differences in sustained level of use, were associated with outcomes in young adulthood. Although some trajectory groups exhibited similar overall directions of change, groups differed meaningfully in their level of digital media use across adolescence, and distinguishing cumulative exposure level rather than slope alone, was central to the study’s research questions. Maximum likelihood estimates were then used to assign participants to trajectory groups based on their highest posterior probability of class membership. Trajectory group membership was treated as a fixed categorical variable in subsequent regression analyses, with the low-use group serving as the common referent. While approach does not explicitly account for classification uncertainty, given that outcomes were assessed cross-sectionally at Wave IV and analyses were exploratory in nature, this approach was selected to facilitate interpretability of associations between trajectory membership and outcomes. Average posterior probabilities for each trajectory group exceeded recommended thresholds, indicating adequate classification quality (i.e., participants with similar digital media use trends over time were grouped together). Using a forward-selection approach, stepwise procedures were used to determine number of trajectory groups and the best-fit model, including linear, quadratic, and cubic growth curve parameters [33,34]. The best-fit model was determined using Bayesian Information Criterion (BIC), parsimony, and interpretability of trajectory patterns, and model parameters were estimated using maximum likelihood estimates, which considered the statistical probability of each participant belonging to a trajectory group and the probability of the observed data given group membership. Prior to these analyses, an outlier analysis was conducted to address extreme values in self-reported digital media use. Reports exceeding approximately 3 SD above the mean (i.e., 84 h/week, equivalent to more than 12 h/day) were treated as missing prior to trajectory estimation. This liberal threshold was selected to allow high levels of digital media use to remain in the sample and excluded values unlikely to reflect daily behavior given typical waking hours. Further, this threshold was selected to reduce the influence of likely misreported values and extreme skewness, which initially affected distributional properties and model estimation. This cutoff resulted in a normal distribution of the originally negatively skewed variable. This affected 820 participants (12.11%) of the original sample. Sensitivity analyses evaluating alternative handling of extreme values yielded similar results, so the primary analytic approach was therefore retained. No other issues were found regarding normality and homogeneity of variance. Participants missing digital media use data in Waves I–IV were also excluded from analyses. Survey weights were utilized to ensure data was nationally representative, and missing data were handled using the MISSING and NOMCAR functions.

Digital media use trajectory groups were used as predictors of Wave IV mental health outcomes including diagnoses of anxiety and depression, depressive symptomology, suicidal ideation, and suicide attempts. Three separate general linear models (PROC SURVEYREG) were used to assess the predictive relationship between media use trajectory groups and depressive symptomology, working memory, and short-term memory. Prior to these analyses, assumptions of normality and homogeneity of variance were assessed. Four separate logistic regressions (PROC SURVEYLOGISTIC) were used to assess the predictive relationship between media use trajectory group and anxiety diagnosis, depression diagnosis, suicidal ideation, and suicide attempts. Logistic regression models accounted for the complex survey design using survey-weighted procedures. Covariates were pre-determined based on the prior literature (Table 1). Prior to these analyses, data were checked for multicollinearity. In addition, a cross-sectional analysis of outcomes at Wave IV was used to assess the longitudinal effects of digital media use at various time points across the transition from adolescence into young adulthood.

Four separate logistic regressions (PROC LOGISTIC) were used to assess the predictive relationship between parental support, family connectedness, face-to-face interactions with peers, and self-esteem with digital media use trajectory group. Prior to these analyses, data were checked for multicollinearity. Covariates were predetermined based on the past literature (Table 1). Specifically, sex, race, age, income, and rurality at Waves I and IV were included as covariates in all models given demographics have been shown to influence digital media use patterns and mental health outcomes. As depression is a well-established risk factor for suicidal ideation and attempts, depression diagnosis and symptomology were also included as covariates in those models. All outcomes were selected a priori based on theory and the prior literature; therefore, formal corrections for multiple comparisons were not applied, and results are interpreted with appropriate caution.

## 3. Results

**Figure 1:**
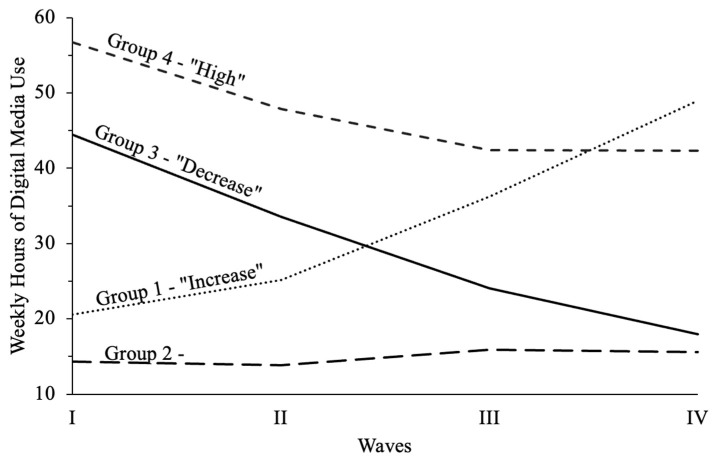
Adolescent digital media use trajectories across Waves I–IV.

The final sample included 6767 adolescents (M_age_ = 15.53, SD = 1.42; 56.86% female, 66.89% White) aged 11–17 and 24–30 at Waves I and IV, respectively. See Table 2 for participant demographic information by trajectory group. Four digital media use trajectory groups emerged (Figure 1). Group 2, “low” included the majority of participants (73.36%). Participants in Group 2 exhibited the lowest digital media use across Waves I–IV, averaging approximately 15 h per week across Waves. Participants in Group 1, “increase” (9.97%), exhibited a steady increase in digital media use across Waves I–IV, whereas individuals in Group 3, “decrease” (13.94%), exhibited a steady decrease in digital media use across Waves I–IV. Group 4, “high”, included the least number of participants (2.73%), and exhibited high levels of digital media use across Waves I–IV. See Table 3 for a summary of weekly hours of digital media use by trajectory group and wave.

Digital media use trajectory group significantly predicted Wave IV short-term memory such that individuals in Group 4 (M = 6.28, SD = 4.01) experienced decreased short-term memory at Wave IV compared to individuals in Group 2 (M = 6.86, SD = 2.94). Of note, adolescents in Group 4 exhibited an average of approximately 47 h of digital media use per week across waves which was much higher than that of other groups. Wave IV digital media use did not independently predict Wave IV short-term memory.

Digital media use trajectory group also significantly predicted suicide attempts such that individuals in Groups 3 had 7% greater odds of a suicide attempt in the past 12 months compared with individuals in Group 2 (OR = 1.07, 95% CI [1.02, 1.34]), and adolescents in Group 4 had 10% greater odds of a suicide attempt in the past 12 months compared with individuals in Group 2 (OR = 1.10, 95% CI [1.01, 1.42). Notably, adolescents in Groups 3 and 4 exhibited the greatest digital media use per week at Waves I and II. Specifically, adolescents in Groups 3 and 4 exhibited 44.44 and 56.73 h per week at Wave I and 33.56 and 47.84 h per week at Wave II, respectively. For comparison, adolescents in Group 2 exhibited 14.34 and 13.84 h per week at Waves I and II, respectively. Wave IV digital media use did not independently predict Wave IV suicide attempts. Neither digital media use trajectory group nor Wave IV digital media use significantly predict Wave IV depressive symptomology, working memory, depression diagnosis, anxiety diagnosis, or suicidal ideation. Wave IV parental support, family connectedness, face-to-face interactions with peers, and self-esteem did not predict digital media use trajectory group membership. See Table 4 and Table 5 for a summary of the results.

## 4. Discussion

Adolescents in Group 4 “high” experienced significantly decreased short-term memory at Wave IV as compared with individuals in Group 2 “low”. However, Wave IV digital media use did not independently predict short-term memory in young adulthood. Taken together, these results suggest that a pattern of high levels of digital media across adolescence may have a compounding effect on short-term memory in young adulthood. While the underlying mechanisms in this relationship are unclear, cross-sectional research posits that negative affect may mediate this relationship [35]. A recent study found that increased digital media use was associated with decreased negative affect and subsequent short-term memory failures [36]. While we did not find a longitudinal relationship between digital media use and negative affect, this may be due to the effects of other, more salient variables present in this relationship (e.g., sleep, physical health). These findings may also be interpreted considering preliminary research demonstrating a relationship between consistently high levels of digital media use—like those exhibited by individuals in Group 4—and anatomical changes in brain cortex grey matter. It is posited that constant, fast access to information via the internet may alter the brain’s need to store facts and knowledge not otherwise gained by personal experiences [24]. Overall, these results support the prior literature on associations between increased digital media use and decreased short-term memory [24,35,36]. These findings may contribute to existing models of cognitive development by suggesting that sustained patterns of high digital media use across adolescence, rather than digital media use at a single time point, may be more relevant for understanding changes in specific cognitive processes such as short-term memory. These results also support the need for (1) further research on other aspects of cognitive ability which may be negatively affected by increased digital media use and (2) further research on anatomical changes due to digital media use.

Suicide attempts were rare in the analytic sample (overall prevalence of 1.25%), and observed odds ratios therefore reflect modest differences in event occurrence across trajectory groups. Although the observed odds ratios were modest in magnitude, adolescents in Groups 3 “decrease” and 4 “high” were at increased odds of a suicide attempt in the past 12 months at Wave IV as compared with individuals in Group 2 “low”. These results suggest that increased levels of digital media use in adolescence and young adulthood may be associated with increased risk of suicide attempts in young adulthood. Cyberbullying has been proposed as a potential pathway linking digital media use and suicidality [21] and a meta-analysis of 11 longitudinal studies found that peer victimization in adolescence consistently predicted suicidal behaviors in young adulthood [37]. While the present study does not allow for direct evaluation of such mechanisms, peer victimization in adolescence may affect later drug use, self-esteem, and interpersonal relationships, which may contribute to susceptibility to suicide attempts [38,39]. Importantly, these findings also suggest that models of mental health may benefit from greater attention to sustained patterns of digital media use across adolescence, rather than relying on digital media exposure assessed at a single time point.

Digital media use trajectory group and Wave IV digital media use did not predict Wave IV depressive symptomology, depression diagnosis, anxiety diagnosis, working memory, or suicidal ideation. These null findings suggest that associations between digital media use and mental health outcomes may not be uniform across domains. While these findings differ from some of the prior cross-sectional literature, this may reflect the influence of other, more salient underlying factors. For example, the positive relationship between sedentary behaviors (e.g., digital media use) and risk for depression is well-documented in research. Increased sedentary behaviors are also consistently associated with increased risk for obesity and poor sleep, which may play a more salient role in the relationship between digital media use and depression. Overall, these results suggest that digital media use may be one of several contextual factors related to mental health, rather than a primary, independent driver of depressive or anxiety-related outcomes.

We further assessed factors in adolescence that may contribute to digital media use patterns across waves. Specifically, we examined parental support, family connectedness, face-to-face interactions with peers, and self-esteem as predictors of trajectory group membership. As none of these variables significantly predicted trajectory group membership, these null findings may suggest that commonly cited interpersonal and individual factors may not independently explain long-term patterns of digital media use. This may be due to measurement fallibility. Data for those variables were obtained via self-report on a 5-point Likert scale. Self-report Likert scales have been associated with response bias [40] and require caution when being interpreted. Alternatively, digital media use trajectories may be shaped by a broader set of contextual, technological, and environmental influences not captured in the present study. Future research should re-assess the measurement of these variables and their relationship with digital media use in adolescence. Future research is also needed to assess other variables that may affect digital media use trends.

This study has several implications. Although effect sizes were modest, the results are concerning given the significant findings between longitudinal patterns of digital media use and suicide attempts and short-term memory in young adulthood. As suicide attempts among adolescents have nearly doubled since 2010 [5], further research is critical to clarify whether and how patterns of digital media use may contribute to suicide risk. Further, interventions aimed at reducing and mitigating the potential effects of digital media use on suicide attempts are needed. For example, teachers, pediatricians, and related personnel may consider discussing digital media use with parents and adolescents within the context of adolescent mental health and well-being. Parents may also consider monitoring or setting boundaries around daily digital media use. Regarding short-term memory, future research is needed to assess underlying mechanisms. In the meantime, schools that are reliant on technology in the classroom may consider balancing screen-based instruction with non-screen-based learning activities to decrease screen-time in the classroom. Furthermore, our results highlight the need for additional research to clarify mechanisms that may underly the relationship between digital media use and depression and anxiety diagnoses, depressive symptomology, working memory, and suicidal ideation.

The strengths of this study include the nationally representative sample and study design, which allowed for examination of pathways linking patterns of digital media use in adolescence with mental health outcomes in young adulthood. Several limitations should also be noted. Digital media use was self-reported across waves, and we were unable to control for time between waves. Further, the broad measures of digital media use used in this study limited our ability to assess qualitative differences in media engagement (e.g., type of content, context of use, purpose of engagement), which may differentially relate to mental health and cognitive outcomes. However, the primary aim of this study was to examine cumulative patterns of digital media use over time, irrespective of media type, content, or purpose of use. Future research should build on this work by integrating cumulative exposure models with more specific assessments of digital media use.

In addition, measures of digital media use differed across Waves I–IV, with earlier waves capturing primarily television, video, and video game use, and more interactive forms of media (e.g., internet) were not assessed until Wave IV. As such, trajectory groups should be interpreted as reflecting patterns of overall digital media exposure across developmental periods rather than specific media behaviors measured repeatedly over time. Notably, data collection for the dataset began in 1994 and concluded in 2009, a period during which digital media primarily consisted of television, videos, video games, and limited internet use. Technological advancements in the past decade have produced new forms of digital media use not accounted for in the present study (e.g., social media, smartphones). As such, the present study’s findings may not directly generalize to modern forms of digital media use. However, the results provide valuable insight into how cumulative exposure to digital media use across developmental periods relates to later mental health and cognitive outcomes, independent of specific technologies, and may inform broader developmental models of digital media use. These findings also underscore the need for future research using more recent cohorts and updated media measures, particularly given the exponential increase in digital media use in recent years.

Furthermore, diagnoses of depression and anxiety were based on self-report rather than clinical interview. Self-report diagnostic data may underestimate or overestimate true prevalence and should be interpreted as indicators of reported clinical history rather than confirmed psychiatric diagnoses. However, self-report diagnoses are commonly used in large-scale longitudinal studies and allow for the examination of population-level associations when structured diagnostic interviews are not feasible.

## 5. Conclusions

In summary, we found that patterns of digital media use in adolescence may contribute to suicide attempts and short-term memory in young adulthood. These results highlight the importance of critically considering the need to educate families and adolescents of the potential risks of high rates of digital media use and potential benefits of reducing screen time throughout adolescence. These results further suggest that models of mental health and cognitive development may benefit from greater attention to sustained patterns of digital media use across adolescence. Non-significant findings highlight the need for additional research aimed at clarifying these relationships, as well as identifying factors in early adolescence which may contribute to digital media use trajectories.

## Figures and Tables

**Table 1:** Predetermined Covariates.

Measure	Covariates
Digital Media Use	Sex Race Age (Wave IV) Income (Wave I) Rurality (Waves I and IV)
Suicidal Ideation	Depression Diagnosis (Wave IV) Depressive Symptomology (Waves I and IV)
Suicide Attempts	Depression Diagnosis (Wave IV) Depressive Symptomology (Waves I and IV)

**Table 2:** Participant Demographics by Trajectory Group.

Group	n	Wave I Age	Race			Sex
		Years	% White	% Black	% Other *	% Male
Group 1 “Increase”	675	15.51	60.73	24.88	14.39	57.56
Group 2 “Low”	4964	15.58	70.31	16.70	12.99	39.11
Group 3 “Decrease”	943	15.29	52.72	35.15	12.13	61.51
Group 4 “High”	185	15.31	55.36	31.14	13.50	15.31

* “% Other” includes individuals who selected Native American, Asian or Pacific Islander, Hispanic, and/or “Other” when reporting race.

**Table 3:** Weekly Hours of Digital Media Use by Trajectory Group and Wave.

Group	Wave I	Wave II	Wave III	Wave IV
Group 1 “Increase”	20.12	25.04	35.32	48.51
Group 2 “Low”	14.34	13.84	15.62	15.94
Group 3 “Decrease”	44.44	33.56	24.08	18.12
Group 4 “High”	56.73	47.84	42.51	42.51

**Table 4:** Summary of Analyses and Results.

**Digital Media Use Trajectory General Linear Models**			
	df	F	*p*
Wave IV Depressive Symptomology	3	1.62	0.19
Wave IV Working Memory	3	0.30	0.82
Wave IV Short-Term Memory	3	8.02	<0.001 *
**Digital Media Use Trajectory Logistic Regression**			
	df	F	*p*
Wave IV Anxiety Diagnosis	3	0.43	0.73
Wave IV Depression Diagnosis	3	1.78	0.15
Wave IV Suicidal Ideation	3	0.63	0.59
Wave IV Suicide Attempts	3	283.30	<0.001 *
**Logistic Regression for Digital Media Use Trajectory**			
	df	F	*p*
Wave I Parental Support	3	1.61	0.19
Wave I Family Connectedness	3	1.70	0.17
Wave I Face to Face Interactions with Peers	3	2.04	0.11
Wave I Self-Esteem	3	1.57	0.22
**Wave IV Digital Media Use General Linear Model**			
	df	F	*p*
Wave IV Depressive Symptomology	2	0.60	0.55
Wave IV Working Memory	2	0.93	0.40
Wave IV Short-Term Memory	3	0.22	0.88
**Wave IV Digital Media Use**			
	df	F	*p*
Wave IV Anxiety Diagnosis	128	−0.94	0.35
Wave IV Depression Diagnosis	128	0.30	0.76
Wave IV Suicidal Ideation	128	−0.58	0.56
Wave IV Suicide Attempts	128	0.09	0.10

* Denotes *p*-value of <0.05. df (degress of freedom) indicate the number of independent pieces of information used to estimate the test statistic. F represents the ratio of explained variance to unexplained variance in the outcome.

**Table 5:** Follow-Up Tests for Significant Differences.

**Wave IV Short-Term Memory Pairwise Group Comparisons**		**Adjusted Mean Difference**	**SE**	***p***
Group 2	Group 1	−0.27	0.14	0.07
Group 2	Group 3	−0.27	0.19	0.17
Group 2	Group 4	−0.58	0.13	<0.001 *
Group 3	Group 4	0.31	0.24	0.19
Group 3	Group 1	0.00	0.22	1.00
Group 4	Group 1	0.31	0.19	0.11
**Wave IV Suicide Attempts Pairwide Odds Ratios**		**OR**	**95% CI**	***p***
Group 2	Group 1	0.70	0.01, 1.40	0.31
Group 2	Group 3	1.10	1.02, 1.34	<0.01 *
Group 2	Group 4	1.07	1.01, 1.42	<0.01 *

* Denotes *p*-value of <0.05. Standard errors (SE) represent the precision of parameter estimates, reflecting the variability of the estimate across repeated samples. Smaller SE values indicate greater precision of the estimated effect.

## Data Availability

The data presented in this study are available on request from the corresponding author. The data are not publicly available due to privacy and ethical reasons.

## Abbreviations

The following abbreviations are used in this manuscript:

Add Health	National Longitudinal Study of Adolescent Health
